# Immune dysregulation in pediatric tic disorders: mechanisms, biomarkers, and therapeutic frontiers

**DOI:** 10.3389/fimmu.2026.1708940

**Published:** 2026-03-10

**Authors:** Xu Sun, Xiaohong Bai

**Affiliations:** 1 First Clinical College of Liaoning University of Traditional Chinese Medicine, Sheyang, Liaoning, China; 2 Affiliated Hospital of Liaoning University of Traditional Chinese Medicine, Shenyang, Liaoning, China

**Keywords:** immune dysregulation, neuroinflammation, PANDAS/PANS, tic disorders, Tourette syndrome

## Abstract

Tic Disorders (TDs) are common neurodevelopmental disorders characterized by complex pathophysiological mechanisms. A growing body of evidence in recent years suggests that immune system dysregulation plays a critical role in the pathogenesis and clinical course of TDs in a subset of pediatric patients. This review aims to systematically summarize the current understanding of the core mechanisms of immune dysregulation in pediatric TDs, potential biomarkers, and related therapeutic frontiers. We detail three core pathophysiological pathways, Post infectious autoimmunity, represented by the Pediatric Autoimmune Neuropsychiatric Disorders Associated with Streptococcal Infections (PANDAS) and Pediatric Acute-onset Neuropsychiatric Syndrome (PANS) models. Its core mechanism involves the production of autoantibodies induced by molecular mimicry, which target basal ganglia neurons, such as cholinergic interneurons and dopamine receptors. Neuroinflammation is another critical pathway. This process involves T helper 17 (Th17) cell-mediated disruption of the blood-brain barrier and microglial activation. It is further characterized by elevated pro-inflammatory cytokines, such as Tumor Necrosis Factor-alpha (TNF-α) and Interleukin-12 (IL-12). Microbiota-gut-brain axis dysregulation, wherein gut dysbiosis and compromised intestinal barrier function influence central nervous system (CNS) function through the neuro immune endocrine network. Building upon this framework, we evaluate potential biomarkers across various dimensions, including the findings and limitations in serology (cytokines), cerebrospinal fluid analysis (oligoclonal bands, MCP-1), neuroimaging (volumetric changes in the basal ganglia and PET imaging of neuroinflammation), and genetics (variations in the IL-1RN gene). Finally, we discuss the evolution from conventional treatments to emerging immune-targeted therapies. This encompasses core immunomodulatory therapies (Intravenous Immunoglobulin (IVIG) and plasmapheresis) and promising future strategies, such as fecal microbiota transplantation (FMT), targeted B-cell therapies, and small-molecule anti-inflammatory drugs. In conclusion, a deeper understanding of the immunological basis of TDs is paving the way for the development of more precise diagnostic tools and novel, individualized immunomodulatory interventions.

## 1 Introduction

Tic Disorders (TDs), including their most severe form, Tourette Syndrome (TS), are neurodevelopmental conditions characterized by involuntary, rapid, and repetitive motor and/or vocal tics (1). These disorders frequently co-occur with psychiatric comorbidities such as Attention-Deficit/Hyperactivity Disorder (ADHD) and Obsessive-Compulsive Disorder (OCD), significantly impacting the quality of life of affected children (2, 3). Historically, the pathophysiological understanding of TDs has centered on genetic susceptibility and dysfunction of the dopaminergic system within the cortico-striato-thalamo-cortical (CSTC) circuitry. It is noteworthy that the natural history of classic Tic Disorders often involves a spontaneous reduction or remission of symptoms by late adolescence. However, the classical dopaminergic model does not fully explain the clinical spectrum. It specifically fails to account for fluctuating symptoms, acute exacerbations, and the environmental triggers observed in some patients (4).

In recent years, research has increasingly expanded to explore the role of the immune system (4). This shift was largely driven by clinical observations of children experiencing abrupt symptom onset following infections, particularly Group A β-hemolytic streptococcus (5). These observations led to the hypothesis of Pediatric Autoimmune Neuropsychiatric Disorders Associated with Streptococcal Infections (PANDAS) and the broader concept of Pediatric Acute-onset Neuropsychiatric Syndrome (PANS) (5, 6). It is important to distinguish that PANS/PANDAS represents a specific clinical subgroup characterized by acute, explosive onset, which differs from the typical, more gradual course of classic TS. Underlying this potential immuno-neuro connection, current research hypothesizes multiple, interconnected biological mechanisms, including post-infectious autoimmunity, neuroinflammation, and dysregulation of the microbiota-gut-brain axis (7, 8).

This review aims to systematically summarize the current understanding of immune dysregulation in pediatric tic disorders. We will elucidate the core proposed pathological mechanisms, ranging from autoimmunity to gut-brain axis dysfunction, while distinguishing between established findings and theoretical models. Building on this framework, we will evaluate the clinical utility and limitations of potential biomarkers in serology, cerebrospinal fluid, and neuroimaging (**Table 1**). Finally, we will discuss therapeutic frontiers, including immunomodulatory interventions and emerging strategies, with a critical assessment of the current evidence base.

**Table 1:** Comparative analysis of potential immune-related biomarkers in tic disorders.

Biomarker Category	Specific Markers	Sample Matrix	Main Findings in Tic Disorders / PANDAS	Reproducibility and Clinical Applicability	Methodological Limitations
Infectious / Serological	ASO, Anti-DNase B	Serum	Elevated titers indicate recent Group A *Streptococcus* (GAS) infection.	High reproducibility for infection detection; low applicability for TD diagnosis (high background rate in healthy children).	Establishes temporal association only; not specific to tic pathophysiology.
Autoantibodies	Anti-D1R, Anti-D2R, Anti-tubulin, Anti-lysoganglioside-GM1	Serum	Increased binding to cholinergic interneurons and dopamine receptors in PANS/PANDAS subgroups.	Low / controversial. Findings are not consistently replicated across cohorts. Currently for research use, not routine diagnosis.	Lack of specificity; antibodies also found in other neurological conditions. Cell-based assay methodologies vary.
Inflammatory Cytokines	IL-6, TNF-α, IL-1β	Serum	Elevated levels suggest a systemic low-grade inflammatory state in some patients.	Variable. Results vary significantly between studies.	Affected by circadian rhythm, stress, medication, and disease fluctuation. Lack of longitudinal data.
Intrathecal Immune Markers	Oligoclonal Bands (OCBs)	CSF	Positive OCBs found in ~20–38% of adult TS patients, suggesting intrathecal antibody synthesis.	Low in pediatrics. Often negative in pediatric cohorts; higher prevalence in adult refractory cases.	Invasive procedure (lumbar puncture). Specific antigen targets remain unidentified.
Neuroinflammation Imaging	TSPO signal (via PET)	Neuroimaging (PET)	Increased binding in basal ganglia and thalamus, indicating microglial activation.	Moderate. Replicated in specific adult cohorts but limited data in children.	Radiation exposure restricts pediatric use. High cost and limited availability. Mainly reflects adult pathology.
Gut Microbiome	↑ Bacteroidetes, ↓ Firmicutes, GABA-degrading bacteria	Stool (feces)	Dysbiosis observed; correlated with tic severity. Reduced GABA-producing species.	Preliminary. Emerging evidence, but high inter-individual variability.	Causality unclear; changes may be secondary to diet, stress, or antipsychotic medications rather than a primary cause.

## 2 The role of the immune system in the pathophysiology of tic disorders

### 2.1 Post infectious autoimmunity: PANDAS and PANS

While classic TDs are primarily understood as complex neurodevelopmental conditions with a strong genetic basis and a typically gradual onset, emerging evidence suggests that post-infectious autoimmune mechanisms may drive pathophysiology in a specific subset of patients. Among these, Pediatric Autoimmune Neuropsychiatric Disorders Associated with Streptococcal Infections (PANDAS) and Pediatric Acute-onset Neuropsychiatric Syndrome (PANS) represent distinct clinical entities. Unlike the fluctuating but generally chronic course of classic Tourette Syndrome, these syndromes are characterized by an abrupt, dramatic onset of symptoms. They serve as a critical clinical model for exploring the immunological origins of neuropsychiatric symptoms in this specific subgroup, rather than explaining the etiology of all tic disorders (5–9).

In recent years, the role of post-infectious autoimmune mechanisms in a subset of pediatric tic disorders and related neuropsychiatric conditions has garnered increasing attention. Among these, Pediatric Autoimmune Neuropsychiatric Disorders Associated with Streptococcal Infections (PANDAS) and Pediatric Acute-onset Neuropsychiatric Syndrome (PANS) provide a critical clinical model for understanding these disorders. These two concepts describe a group of clinical entities characterized by the acute onset of neuropsychiatric symptoms, the pathophysiology of which is believed to be closely associated with immune system dysregulation. They serve as an important theoretical framework for exploring the immunological origins of pediatric tic disorders and related neuropsychiatric symptoms (5–9). Due to clinical heterogeneity and small sample sizes, the current pathophysiological models are mainly based on preliminary observations and still require validation through large-scale cutting-edge research.

#### 2.1.1 The PANDAS hypothesis: an autoimmune model linked to Streptococcal infections

PANDAS, an acronym for “Pediatric Autoimmune Neuropsychiatric Disorders Associated with Streptococcal Infections,” was first described in 1998 (5). This hypothesis defines a specific clinical subgroup whose core features are the abrupt, dramatic onset of OCD and/or tic disorders, with a temporal association between the onset or exacerbation of these symptoms and a Group A β-hemolytic streptococcal (GABHS) infection. The proposition of PANDAS was based on its clinical and pathomechanical similarities to Sydenham’s chorea (SC), the latter of which has been confirmed as a post-GABHS autoimmune disorder targeting the basal ganglia (10, 11). According to the original definition, a diagnosis of PANDAS requires meeting the following five criteria, Core Symptoms: Presence of a diagnosis of OCD and/or a tic disorder. Pediatric onset: The initial appearance of symptoms occurs between the age of 3 and the prepubertal period. Episodic course: The symptoms follow an episodic course, characterized by a sudden, acute onset and significant exacerbations, accompanied by periods of remission. Association with GABHS: There is a clear temporal relationship between the emergence or worsening of symptoms and a GABHS infection. Neurological Abnormalities: The presence of associated neurological abnormalities, such as motor coordination problems or adventitious involuntary movements. The pathophysiological hypothesis of PANDAS is primarily centered on the mechanism of “molecular mimicry”. This hypothesis posits that antibodies produced by the body in response to a GABHS infection not only recognize streptococcal antigens but also cross-react with and attack the host’s central nervous system, specifically targeting neuronal autoantigens in the basal ganglia region. This process leads to neuronal dysfunction and the manifestation of psychiatric and behavioral symptoms (7).

#### 2.1.2 PANS: a broader concept of acute-onset neuropsychiatric syndrome

As research has progressed, a significant number of cases have been clinically observed that meet the core clinical features of PANDAS (acute onset of neuropsychiatric symptoms) but for which a definitive association with a GABHS infection cannot be confirmed. To encompass this patient population and to account for the possibility that other pathogens or non-infectious factors could trigger similar symptoms, the broader diagnostic concept of PANS (Pediatric Acute-onset Neuropsychiatric Syndrome) was proposed in 2012 (12). The diagnostic criteria for PANS shift the emphasis away from a specific pathogenic trigger and instead focus on the core features of the clinical presentation: Core symptoms: The abrupt, dramatic onset of OCD or severely restrictive food intake (9). Concurrent symptoms: The simultaneous acute onset of at least two of the following seven categories of additional neuropsychiatric symptoms: Anxiety Emotional lability and/or depression. Irritability, aggression, and/or severe oppositional behaviors. Behavioral (developmental) regression. Deterioration in school performance. Sensory or motor abnormalities. Somatic signs and symptoms, such as sleep disturbances, enuresis, or urinary frequency. Exclusion Criteria: The symptoms are not better explained by another known neurological or medical disorder, such as Sydenham’s chorea, systemic lupus erythematosus, or Tourette syndrome.

The introduction of the PANS concept holds significant clinical importance. Firstly, it redirects the diagnostic focus from “searching for evidence of a streptococcal infection to identifying the clinical pattern of acute onset”, thereby preventing missed diagnoses in cases where the infectious detection window has passed. Secondly, it acknowledges that, in addition to GABHS, a variety of other pathogens (Mycoplasma pneumoniae, influenza virus, coxsackievirus) and even non-infectious stressors (psychological stress) may be potential triggers for the syndrome. Notably, the diagnostic criteria for PANS have moved tics out of the core symptoms to better differentiate it from Tourette syndrome, which typically presents with a more subacute onset.

#### 2.1.3 Clinical distinctions and controversies

It is crucial to distinguish PANDAS/PANS from classic tic disorders clinically. The hallmark of PANDAS/PANS is the “explosive” onset of symptoms (reaching maximal severity within 24 to 48 hours), often accompanied by severe separation anxiety, emotional lability, or urinary symptoms, which contrasts with the waxing and waning course typically seen in classic TS.

Despite the utility of the PANDAS/PANS hypothesis, significant academic controversy persists regarding its diagnostic validity (13). Critics argue that the diagnostic criteria rely heavily on clinical history and the temporal association with infections, which can be subjective and prone to recall bias. Furthermore, a definitive causal link between streptococcal infection and tic onset has not been universally replicated in large epidemiological studies. Unlike classic TS, where genetic factors are prominent, the PANDAS/PANS model emphasizes environmental triggers (infections) acting upon a susceptible immune background. Therefore, these conditions should be viewed as a distinct, immune-mediated subgroup, and findings from this population cannot be automatically generalized to the broader population of children with tic disorders.

### 2.2 Core mechanism 1: molecular mimicry and autoantibodies

#### 2.2.1 Molecular mimicry

Within the PANDAS/PANS framework, the leading immunopathological hypothesis is the mechanism of molecular mimicry, which is considered the critical bridge linking the initial infection to the subsequent neuropsychiatric symptoms. This model proposes that antibodies produced to clear pathogens, particularly GABHS, mistakenly attack neuronal targets. This cross-reaction occurs due to structural similarities between pathogenic antigens and the host’s CNS proteins. This targeting is particularly directed at the basal ganglia region.

**Figure 1:**
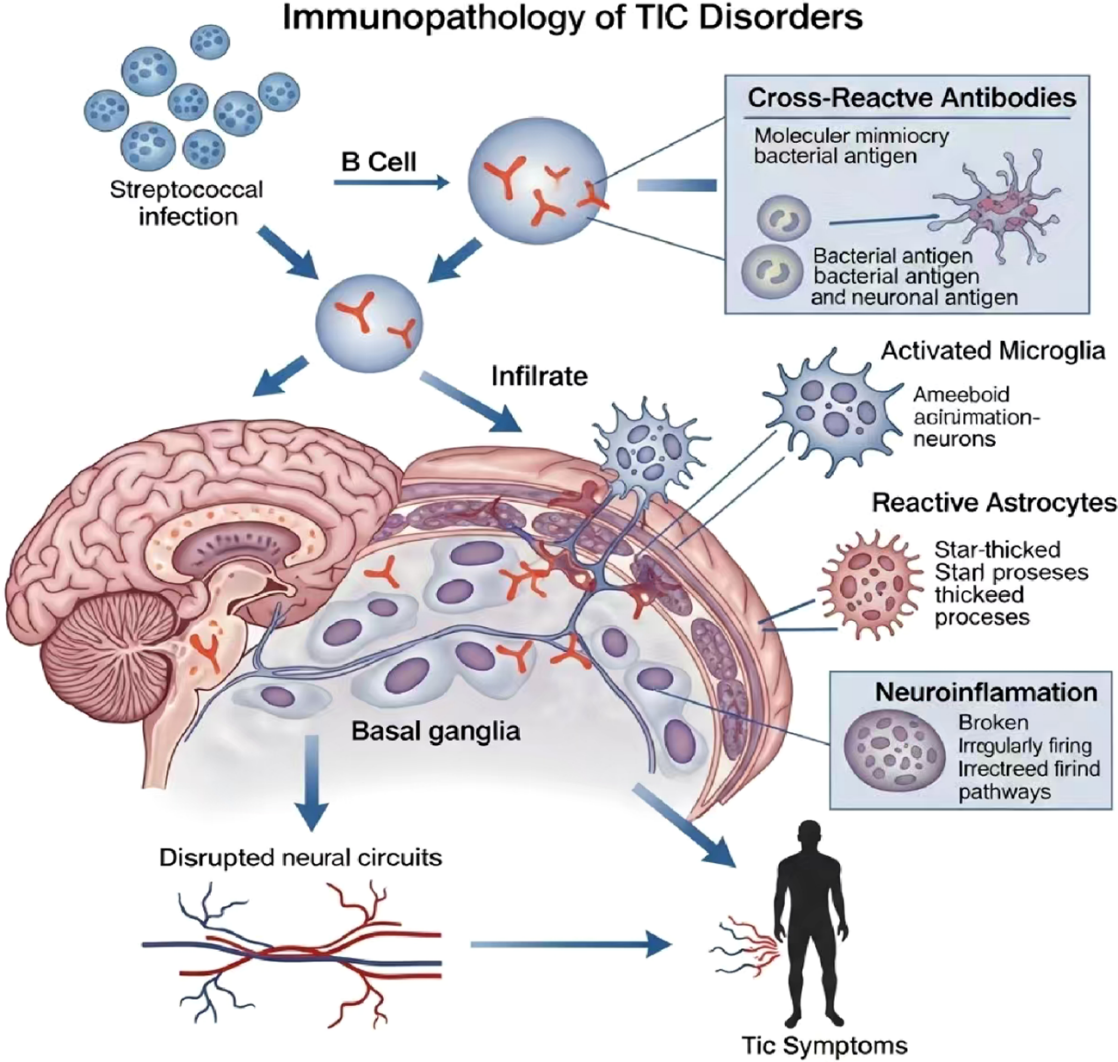
The immunopathology of tic disorders. This schematic depicts the immunopathological cascade initiated by a peripheral infection (Streptococcus) that leads to tic symptoms. (1) A peripheral infection activates B cells, leading to antibody production. (2) Through a mechanism of molecular mimicry, these antibodies cross react, recognizing not only bacterial antigens but also self-neuronal antigens in the host’s central nervous system, particularly within the basal ganglia. (3) These autoantibodies and immune cells infiltrate the brain parenchyma, a process potentially facilitated by a compromised blood-brain barrier. (4) Within the CNS, this triggers a state of neuroinflammation, characterized by the activation of resident immune cells, including microglia and astrocytes. (5) The combination of neuroinflammation and antibody-mediated damage leads to the dysfunction of neural circuits, especially the cortico striato thalamo cortical (CSTC) circuitry. Ultimately, these neuropathological processes manifest clinically as tic symptoms.

Research has demonstrated that antibodies targeting the primary carbohydrate epitope of streptococcus (N-acetyl-β-D-glucosamine) are capable of cross-reacting with neural cells. These cross-reactive autoantibodies are believed to traverse the blood brain barrier (BBB), which has increased permeability due to inflammation, and act directly on the basal ganglia, thereby triggering neuronal dysfunction (14) (**Figure 1**). This process is analogous to the well-established pathogenesis of Sydenham’s chorea, a complication of rheumatic fever, which involves antibody-mediated dysregulation of neuronal cell signaling (15).

#### 2.2.2 Specific autoantibodies targeting the basal ganglia

A series of autoantibodies targeting specific neural substrates has been detected in patients with PANS/PANDAS. The presence of these antibodies provides support for the autoimmune hypothesis of the disorder, although their precise pathogenic role continues to be investigated. One of the most rigorous studies to date found that serum autoantibodies from children with PANS/PANDAS specifically bind to cholinergic interneurons (CINs) in the striatum at significantly higher levels than in control subjects. This binding suggests potential functional consequences; the study indicated that these antibodies might alter and reduce the functional activity of CINs, thereby potentially disrupting the normal function of the cortico-striatal circuitry. Importantly, the level of this antibody binding was found to correlate with the severity of clinical symptoms, and following treatment with intravenous immunoglobulin (IVIG), symptomatic improvement paralleled a decrease in antibody binding levels (16).

Autoantibodies against dopamine receptors D1R and D2R have been described in patients with PANS/PANDAS. These receptors are predominantly distributed in brain regions closely associated with motor and emotional regulation, such as the striatum, substantia nigra, and hippocampus. Mechanistically, anti-D1R/D2R antibodies are thought to suggest dysfunction and dysregulation of the dopamine signaling system by directly internalizing dopamine receptors from the cell surface. In addition to dopamine receptors, autoantibodies against tubulin and lysoganglioside-GM1 have also been detected in PANS/PANDAS patients. These antibodies, along with anti-D1R/D2R antibodies, are part of the commercially available “Cunningham Panel”, a test designed to aid in the diagnosis and treatment monitoring of PANS/PANDAS. Research in rheumatic fever-associated chorea has also identified tubulin as an important neuronal target of autoantibodies (6, 17). Although the Cunningham Panel has been commercialized, its effectiveness in clinical diagnosis and long-term disease course monitoring has not yet been widely recognized by the academic community, and its results should be interpreted with caution.

Upon binding to neuronal targets, autoantibodies can further amplify their pathological effects by activating downstream intracellular signaling pathways. Studies have found that the activity of Ca^2+^/calmodulin-dependent protein kinase II (CaMKII) is significantly elevated in the serum and cerebrospinal fluid of patients during the acute phase of PANDAS. This finding is highly specific, as a significant increase in CaMKII activity was not observed even in healthy controls with similarly high antibody levels, suggesting that CaMKII activation may be a critical step in translating antibody binding into neuroinflammation and clinical symptoms. Increased CaMKII activity can directly affect neuronal function, for instance, by causing alterations in dopamine release. The importance of CaMKII is also supported by clinical observations: in a retrospective study, the improvement of patients’ neuropsychiatric symptoms was closely correlated with post-treatment reductions in both autoantibody titers and CaMKII activity (15).

In summary, autoantibodies induced by molecular mimicry targeting a variety of neuronal proteins in the basal ganglia (such as proteins on the surface of CINs, dopamine receptors, and tubulin) constitute a core element of the immunopathology of PANDAS/PANS. They exert their effects by directly interfering with neuronal function and activating downstream signaling pathways, such as CaMKII (18).

### 2.3 Core mechanism 2: neuroinflammation and microglia

A growing body of evidence suggests that neuroinflammation is a key component in the pathophysiology of TDs, with microglial activation playing a central role. As the resident immune cells of the CNS, microglia are not only important participants in neurodevelopment and synaptic pruning, but their abnormal activation is also closely linked to the onset and progression of various neuropsychiatric disorders.

#### 2.3.1 Microglial activation

As the innate immune cells of the CNS, microglia play a critical role in the pathological process of PANDAS/PANS. Microglia are the “sentinels” of the CNS; in a homeostatic state, they constantly monitor their surrounding microenvironment with their motile processes. Under stimuli such as stress, infection, or autoimmunity, microglia can be rapidly activated, transforming from a resting (ramified) state to an amoeboid activated state (19). Activated microglia are considered a primary source of neuroinflammation and may profoundly affect neuronal survival, function, and synaptic plasticity by releasing a series of cytokines, chemokines, and reactive oxygen species (ROS). Evidence supporting microglial activation has been observed in animal models of tic disorders and in imaging studies of patients. For instance, a study using positron emission tomography (PET) found a significant increase in the signal of the translocator protein (TSPO), which is associated with microglial activation, in the basal ganglia region of adult patients with tic disorders, suggesting a state of persistent neuroinflammation in this brain area (20). Furthermore, a murine model of autoimmune neuropsychiatric disorders induced by streptococcal infection (PANDAS) showed that repeated infections could induce significant microglial activation in the basal ganglia. This provides experimental evidence suggesting a link between infection-triggered immune dysregulation and tic-like behaviors (21).

#### 2.3.2 Pro-inflammatory cytokines and their impact on neuronal function

Activated microglia are the primary source of pro-inflammatory cytokines. In children with tic disorders, several studies have examined changes in cytokine levels in peripheral blood and cerebrospinal fluid (CSF). Tumor necrosis factor-alpha (TNF-α), interleukin-6 (IL-6), and interleukin-1β (IL-1β) are among the most studied molecules. A systematic review indicated that serum levels of IL-6 and TNF-α were significantly elevated in patients with tic disorders compared to healthy controls, suggesting a systemic low-grade inflammatory state (22). Although studies on CSF are relatively few and the results are inconsistent, some studies have detected elevated levels of IL-1β and IL-6 in the CSF of some patients, which more directly reflects the inflammatory environment within the CNS (23).

These cytokines are not merely markers of inflammation; they have a direct regulatory effect on the function of neural circuits. For example, TNF-α and IL-1β have been shown to directly influence glutamatergic and GABAergic neurotransmission. High levels of TNF-α can enhance neuronal excitability by regulating the phosphorylation and membrane trafficking of AMPA receptors, while IL-1β can affect the function of NMDA receptors, thereby altering the threshold for synaptic plasticity, such as long-term potentiation (LTP) (24). In the CSTC circuit, which is closely associated with tic disorders, this cytokine-mediated imbalance in neurotransmission may be one of the core mechanisms leading to involuntary movements and sensory filtering deficits.

#### 2.3.3 Disruption of the blood-brain barrier

The Blood-Brain Barrier (BBB) is a physical and biochemical barrier composed of brain microvascular endothelial cells, pericytes, and astrocyte end-feet, which strictly limits the entry of peripheral substances into the CNS (25, 26). In states of persistent systemic or local CNS inflammation, the integrity of the BBB may be compromised. Pro-inflammatory cytokines released by microglia and peripheral immune cells, such as TNF-α and IL-1β, have been shown to increase BBB permeability by downregulating the expression of tight junction proteins (claudin-5, occludin) and upregulating the activity of matrix metalloproteinases (MMPs) (27, 28).

Increased BBB permeability creates a vicious cycle by allowing peripheral immune cells (such as Th17 cells), autoantibodies, and inflammatory molecules to penetrate the CNS. This influx further exacerbates microglial activation and the neuroinflammatory response (29). In patients with tic disorders, while direct research data on BBB integrity is still limited, some studies have found that the presence of infection-related autoantibodies (such as anti-dopamine D2 receptor antibodies) itself implies that the BBB may have functional “leaks”. For these peripherally derived antibodies to act on deep brain structures like the basal ganglia, they must cross a BBB with increased permeability. Therefore, BBB dysfunction suggest by systemic inflammation may be a key bridge connecting peripheral immune dysregulation with CNS pathology.

### 2.4 Core mechanism 3: the gut-brain axis and the microbiome

**Figure 2:**
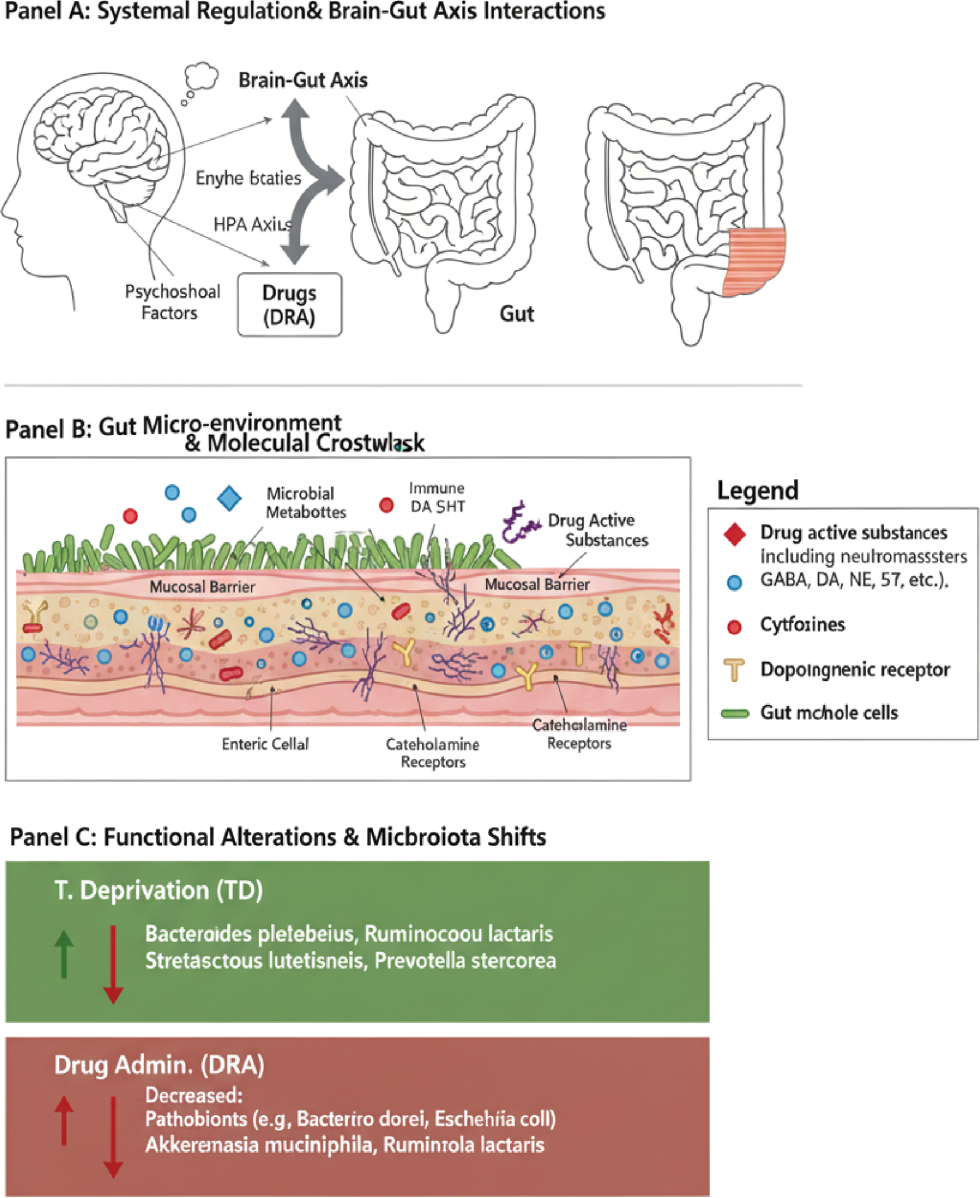
Schematic of the microbiome-gut-brain axis in the pathophysiology of tic disorders. This figure illustrates the complex bidirectional communication network between the gut microbiota and the central nervous system. **(A)** Systemic Regulation & Brain-Gut Axis Interactions. The brain and gut interact through neural (vagus nerve), endocrine (Hypothalamic-Pituitary-Adrenal HPA axis), and immune pathways. External factors, such as psychosocial stress and pharmacological interventions (Drug Administration, DRA), can modulate this axis. **(B)** Gut Micro-environment & Molecular Crosstalk. Gut dysbiosis and compromised intestinal barrier function (leaky gut) can allow microbial metabolites, bacterial components, and drug-active substances to enter systemic circulation. This triggers local and systemic immune responses (cytokine release) and influences the enteric nervous system by acting on catecholamine receptors and modulating neurotransmitters like dopamine (DA) and serotonin (5-HT). **(C)** Functional Alterations & Microbiota Shifts. This panel shows representative changes in microbiota composition under different conditions, such as a specific deprivation state (T. Deprivation) or following drug administration (DRA). 5-HT, serotonin; DA, dopamine; DRA, Drug Administration; GABA, gamma-aminobutyric acid; HPA, Hypothalamic-Pituitary-Adrenal; NE, norepinephrine.

In recent years, the Microbiome-Gut-Brain Axis, a complex communication network linking the gut microbiota, the intestinal barrier, the immune system, and the CNS, has gained increasing attention in the pathophysiology of TDs (30, 31) (**Figure 2**). Dysregulation of this axis is considered an important factor affecting neurodevelopment, neuroinflammation, and behavioral phenotypes. Emerging evidence links TDs to abnormalities in gut microbiome composition and impaired intestinal barrier integrity. These factors may influence brain function through multiple pathways, contributing to the onset and progression of the disorder (32–34).

#### 2.4.1 Gut dysbiosis: microbiome composition changes in children with tic disorders

The gut microbiota of children with tic disorders shows significant compositional imbalance (Dysbiosis). Several studies have consistently revealed differences in microbiota structure between TD patients and healthy controls. At the phylum level, a study by Wang found that the relative abundance of Firmicutes in the feces of children with TDs was significantly lower than in healthy children, while the abundance of Proteobacteria was significantly higher, indicating a change in the macroscopic structure of the microbiota (35).

At the genus and species levels, different studies have observed more specific microbial changes. One study reported that in drug-naïve TD children, the abundance of Bacteroides and Ruminococcus was significantly increased, while the abundance of Prevotella copri from the genus Prevotella and species from the genus Eubacterium was significantly decreased. Notably, specific bacterial species are associated with disease subtypes or severity. For example, the abundance of Ruminococcus was particularly elevated in children with Tourette Syndrome (TS), whereas another group of children with chronic tic disorder showed elevated levels of *Bacteroides* vulgatus. Furthermore, the abundance of Prevotella was found to be negatively correlated with tic severity (YGTSS score) (31, 36).

Pharmacological treatment appears to have a regulatory effect on this dysbiosis. Studies have shown that the gut microbiota composition of TD children treated with dopamine receptor antagonists did not differ significantly from that of healthy children, suggesting that medication may, through some mechanism, promote the restoration of the disturbed microbiota towards a healthy state (37). Animal experiments also provide evidence for the link between gut dysbiosis and TDs. After transplanting fecal microbiota from TS mice to healthy mice via fecal microbiota transplantation (FMT), the recipient mice exhibited similar changes in microbiota structure as the TS mice, such as decreased levels of Firmicutes and Actinobacteria and increased levels of Bacteroidetes and Proteobacteria. This suggests that the dysbiotic features associated with TDs may be “transmissible” (38, 39).

#### 2.4.2 Impaired intestinal barrier function/”leaky gut”

The intestinal mucosal barrier is the body’s physical and immune defense line, and its integrity is crucial for maintaining the homeostasis of the gut-brain axis. In the context of TDs, impaired intestinal barrier function (leaky gut) is hypothesized to be a key link connecting intestinal events with CNS pathology. The disruption of the intestinal barrier allows bacterial components (such as lipopolysaccharide, LPS), metabolites, or incompletely digested food antigens, which are normally confined to the intestinal lumen, to enter systemic circulation (31–40). This can trigger or exacerbate systemic low-grade inflammation and may affect the CNS via the blood-brain barrier.

Epidemiological studies provide clues to the association between gut health and TDs. A population-based study in Taiwan, China, found a significant association between a history of enterovirus infection and the development of TDs, suggesting that immune-inflammatory responses triggered by intestinal pathogens may be a contributing factor in some TD cases. Moreover, gut dysbiosis itself can directly damage the barrier. For example, certain bacteria can degrade the protective mucus layer on the mucosal surface or induce local inflammation, weakening the tight junctions between epithelial cells. At the same time, studies have found that medications used to treat TDs may also affect the intestinal barrier; for example, aripiprazole has been reported to alter intestinal mucosal permeability. This barrier dysfunction, suggest by endogenous or exogenous factors, collectively increases the risk of harmful substances entering the bloodstream, thereby altering the signaling of the gut-brain axis and potentially becoming an important factor driving the neuropathological processes of TDs.

#### 2.4.3 Communication pathways of the microbiome-gut-brain axis

The gut microbiota communicates with the brain bidirectionally through multiple parallel pathways, and dysregulation of these pathways is closely related to the neurobiological mechanisms of TDs (34).

The gut microbiota is a key regulator of neurotransmitter synthesis and metabolism. Research shows that various bacteria in the gut can directly produce or influence the levels of key neurotransmitters (41). For example, Lactobacillus and Bifidobacterium are involved in GABA synthesis, while Streptococcus and Enterococcus are associated with serotonin and dopamine levels. In children with TDs, disturbances in GABA metabolism are particularly prominent. Studies have found that the abundance of Klebsiella pneumoniae, which can degrade GABA, is positively correlated with the worsening of tic symptoms. Conversely, the abundance of GABA-producing bacteria such as Eubacterium, Bifidobacterium, and Akkermansia muciniphila is significantly negatively correlated with YGTSS scores (42). This suggests that microbiota-mediated imbalance in the GABAergic system may be a core pathological link in TDs. Additionally, microbial metabolites, such as short-chain fatty acids (SCFAs), act as important signaling molecules that can cross the blood-brain barrier to directly influence nervous system function and immune signaling (43).

Furthermore, the gut microbiota is a key shaper of the development and functional maturation of the host immune system. Dysbiosis of the gut microbiota can lead to impaired maturation and abnormal function of immune cells within the CNS (such as microglia and astrocytes), thereby compromising the immune surveillance function of the CNS (44). As mentioned earlier, a compromised intestinal barrier allows bacterial products to enter circulation, activating a systemic immune response and generating a large number of inflammatory factors (such as cytokines and chemokines). These circulating inflammatory factors can cross the blood-brain barrier and induce neuroinflammation within the CNS, a process considered an important pathological basis for many neuropsychiatric disorders, including TDs (42). It has been found that certain species of Ruminococcus and Bacteroides, which are enriched in untreated TD children, are thought to be associated with autoimmune diseases and may participate in oxidative stress and promote the production and aggregation of inflammatory factors through their metabolites. The Vagus Nerve is a direct physical pathway connecting the gut and the brain, forming a rapid communication channel for the gut-brain axis. Neurons within the enteric nervous system (ENS) can sense chemical signals from the gut microbiota and their metabolites and transmit this information via vagal afferent fibers to the CNS, thereby influencing brain function, mood, and behavior (45). Although direct research on the vagal pathway in TDs is still in its early stages, this pathway is undoubtedly an important direction for future understanding of how the microbiota can rapidly influence tic symptoms.

In summary, gut dysbiosis, impaired intestinal barrier function, and the resulting abnormal immune, metabolic, and neural signaling collectively constitute the core role of the microbiome-gut-brain axis in the pathophysiological mechanisms of tic disorders. These findings not only deepen our understanding of the etiology of TDs but also provide a solid theoretical basis for developing innovative prevention and treatment strategies based on modulating the gut microecology (such as probiotics, fecal microbiota transplantation) (46).

## 3 Biomarkers for immune-mediated tic disorders

### 3.1 Serological markers

Serological analysis provides an accessible tool for identifying systemic immune dysregulation, though specificity remains a challenge. Elevated titers of anti-streptolysin O (ASO) and anti-deoxyribonuclease B (Anti-DNase B) are hallmark indicators of recent Group A Streptococcus (GAS) infection. However, these markers are not specific to tic disorders and are frequently found in the healthy pediatric population. Their utility lies primarily in establishing a temporal association with infection rather than diagnosing the tic disorder itself (5, 47).

Regarding autoimmunity, research has investigated anti-neuronal autoantibodies against basal ganglia targets, including dopamine receptors (D1R, D2R), tubulin, and lysoganglioside-GM1. Some studies utilizing cell-based assays or the commercially available Cunningham Panel have reported elevated CaMKII activity and specific antibody titers in patients with PANS/PANDAS. However, these findings have not been consistently replicated. Other studies have failed to find significant differences in anti-D2R antibodies between patients and controls, or have found them in other neurological conditions. Consequently, the presence of these antibodies is currently considered an research finding regarding potential pathophysiological mechanisms, rather than a confirmed diagnostic biomarker (48).

Similarly, while peripheral cytokine profiles (elevated IL-6, TNF-α) suggest a pro-inflammatory state in some cohorts, results vary significantly between studies, likely due to the fluctuating nature of the disease and methodological differences (49).

### 3.2 Cerebrospinal fluid markers

Cerebrospinal fluid is in direct contact with the central nervous system, providing a unique window for studying the neuroimmune mechanisms of tic disorders (TD). Compared to peripheral blood, CSF analysis can more directly reflect the immune status and inflammation levels within the CNS. However, due to the invasive nature of lumbar puncture, conducting large-scale CSF studies in children presents ethical and practical difficulties, resulting in relatively scarce data. Nevertheless, existing research has revealed several important clues about central immune dysregulation in TS (50).

#### 3.2.1 Oligoclonal bands and IgG Index

Oligoclonal bands are a marker of intrathecal immunoglobulin G (IgG) synthesis and are commonly used to diagnose inflammatory or autoimmune diseases of the CNS (51). In the healthy population, the positivity rate for CSF OCBs is very low (about 3-5%). However, several studies on TS patients have found a significantly higher positivity rate for OCBs in their CSF. One study of 21 TS patients (average age 29) showed that 38% (8/21) had pathological OCBs (6 positive, 2 borderline) (52). Another prospective study of 20 adult GTS patients confirmed this finding, with a 20% (4/20) positivity rate for OCBs (type 2). Combining these two independent studies conducted in the same laboratory, the overall positivity rate for OCBs in TS patients reached 29% (12/41), strongly suggesting the presence of a sustained humoral immune response within the CNS in some TS patients. It is noteworthy that despite the positive OCBs, the IgG index (a quantitative measure of intrathecal IgG synthesis) was generally normal in these studies, indicating that the immune response in TS may be more localized and qualitative rather than widespread and involving massive IgG production (53, 54). However, the finding of OCBs has not been consistent across all TS cohorts. In some small-sample studies focusing on pediatric patients, no positive OCBs were detected. This discrepancy may reflect the heterogeneity of pathophysiological mechanisms in TS at different age stages or in different subtypes. The study by Wenzel, also found that in a pair of monozygotic twins, only one was positive for OCBs, suggesting that environmental factors may play an important role in triggering a central immune response. Although the discovery of OCBs provides support for central immune activation in a subset of patients, the lack of consistency across pediatric cohorts suggests that intrathecal antibody synthesis is not a universal feature of TDs and may be restricted to specific inflammatory subtypes.

#### 3.2.3 Cytokines and autoantibodies in CSF

In addition to OCBs, the direct detection of cytokines and autoantibodies in CSF provides more specific information about the neuroinflammatory state in TS (55). A case series of 4 male adolescent severe TS patients admitted for sudden symptom exacerbation was the first to report significantly elevated levels of Monocyte Chemoattractant Protein-1 (MCP-1) in their CSF, while 33 other cytokines and chemokines were within the normal range. MCP-1 is a potent chemoattractant that recruits monocytes, memory T cells, and microglia, playing a key role in various CNS inflammatory and neurodegenerative diseases (53). Research suggests that MCP-1 may regulate neuronal excitability by affecting the release of dopaminergic and glutamatergic neurotransmitters, which could be directly related to the pathology of TS. This finding suggests that MCP-1-mediated neuroinflammation may hold an important position in the pathogenesis of TS (56).

In contrast, a case-control study of 5 pediatric TS patients with markers of streptococcal infection did not find significant abnormalities in the frequency of several key chemokines (such as CXCL13, CXCL10), B-cell activating factors (BAFF, APRIL), or lymphocyte subsets (B cells, T cells, NK cells) in their CSF. Although the sample size was small, its results suggest that in certain specific TS subgroups, there may not be widespread immune cell infiltration mediated by classic chemokines. Although the presence of OCBs implies the existence of specific intrathecal antibodies, current screening for known nervous system autoantibodies has mostly yielded negative results. Several studies have systematically tested for a range of known neuronal surface antibodies in the CSF or serum of TS patients, including anti-NMDA receptor, CASPR2, LGI1, AMPA receptor, and GABAB receptor antibodies, with all results being negative. The study by Zhongling also tested for 14 neuro-autoantibodies in the CSF of 4 pediatric patients, and all were negative. Furthermore, non-specific antibody screening using tissue sections and differentiated neuroblastoma cell lines has failed to identify a consistent, specific anti-neuronal antibody binding pattern in the CSF of TS patients (42, 57).

CSF studies reveal a complex immune picture in TS. On one hand, the positive finding of OCBs in some patients and the recent report of elevated MCP-1 levels provide direct evidence for the hypothesis of central nervous system immune dysregulation and neuroinflammation in TS. On the other hand, a specific autoantibody clearly associated with TS has yet to be identified. This suggests that the immune mechanism of TS may involve yet-unidentified antigen targets or a more complex, non-antibody-mediated immune process. Future research will require larger, precisely stratified clinical cohorts combined with more advanced, unbiased detection technologies to uncover the key immune molecules and pathways driving the pathology of TS (58).

### 3.3 Neuroimaging markers

Neuroimaging technologies provide a unique window for non-invasively exploring the neurobiological mechanisms behind Tic Disorders (TD). Although conventional clinical imaging studies (such as T1- or T2-weighted MRI) typically show no obvious abnormalities in children with tic disorders, various advanced imaging techniques, including structural magnetic resonance imaging (sMRI), functional magnetic resonance imaging (fMRI), and molecular imaging (like PET), have revealed specific changes in brain structure, function, and molecular levels associated with the disease (59, 60). These findings are primarily centered on the dysfunction of the CSTC circuit, which is considered central to the pathophysiology of tic disorders.

Brain volumetric analysis using sMRI has consistently found structural abnormalities in the CSTC circuit of patients with tic disorders (61). The most robust finding is a reduction in the volume of the caudate nucleus and putamen (components of the basal ganglia), especially during childhood, a change that tends to normalize or reverse in adulthood (1). These volumetric changes may reflect abnormal neurodevelopmental processes, which could be influenced by early immune challenges or neuroinflammatory processes. Functional magnetic resonance imaging (fMRI) has further revealed abnormalities in dynamic information processing within the CSTC circuit and its connections with other brain regions. Event-related fMRI studies have precisely mapped the sequence of neural activity before and after a tic occurs. Studies have found that about 2 seconds before a tic, the supplementary motor area (SMA) and primary motor cortex begin to activate. Then, 1 second before the tic, the activation expands to regions including the anterior cingulate cortex, putamen, insula, amygdala, and cerebellum. Finally, at the moment of the tic, the thalamus, central operculum, somatosensory cortex, and primary motor cortex reach peak activity. This sequence of activation clearly demonstrates the temporal dynamics of the CSTC circuit involved in tic generation (62).

Resting-state fMRI studies have found that the brain functional networks of children with tic disorders exhibit an “immature” connectivity pattern, characterized by weakened long-range connections and strengthened short-range connections. Specifically, within the CSTC circuit, increased functional connectivity between the putamen and the motor cortex has been observed, and the degree of this enhancement is positively correlated with tic severity (60). Furthermore, functional connectivity between the insula and the SMA is also significantly enhanced and is correlated with the severity of premonitory urges. These findings indicate that even in a resting state without performing a specific task, abnormal functional integration within the CSTC circuit persists, constituting a core functional imaging feature of tic disorders (63, 64).

Additionally, PET provides a powerful tool for visualizing molecular processes *in vivo*. Using radioligands targeting the 18-kDa TSPO, neuroinflammation can be directly assessed, as TSPO is upregulated in activated microglia and astrocytes. PET studies using second-generation TSPO ligands (such as ¹¹CPBR28) have shown evidence of microglial activation in the thalamus and sensorimotor cortex of adult patients with Tourette’s syndrome. This provides direct evidence for a persistent neuroinflammatory process within key nodes of the CSTC network (42).

### 3.4 Genetic markers

A growing body of evidence suggests that immune system dysregulation may play a key role in the pathogenesis of TDs. Factors such as neuroinflammation, maternal immune activation, and dysregulated interactions between the immune system and the brain are all considered potential pathophysiological bases for TDs. Therefore, genetic variation studies focusing on immune function-related genes have become an important direction for uncovering susceptibility to TDs and searching for potential biomarkers. This section aims to review genetic markers of the immune system associated with TDs, with a focus on gene variations related to immune function (65).

The HLA system is the cornerstone of immune regulation, and its gene polymorphisms have been associated with various autoimmune diseases. Association studies in tic disorders and PANDAS have investigated specific HLA class I and class II alleles. Although study results have been inconsistent, some have reported an increased frequency of specific alleles (such as HLA-DRB1*11 and DRB4) in patients. This suggests that the way certain MHC class II molecules present antigens may predispose individuals to post-infectious, autoimmune-mediated CNS sequelae (66, 67). Polymorphisms in genes encoding key components of the immune response are thought to be related to susceptibility to tic disorders. For example, genetic variations in cytokines (such as TNF-α), their receptors, and signaling molecules in inflammatory pathways can alter an individual’s inflammatory threshold and response to infection. Similarly, polymorphisms in Toll-like receptor (TLR) genes, which are crucial for recognizing pathogens, may affect the intensity and nature of the body’s immune response to triggers like GAS, potentially linking innate immunity to the pathophysiology of tics (68).

## 4 Current and emerging therapeutic frontiers

### 4.1 Brief overview of conventional treatments and their limitations

Conventional treatment strategies for pediatric tic disorders primarily revolve around two pillars: behavioral therapy and pharmacological intervention. However, both approaches have certain limitations, prompting the academic community to continuously explore new therapeutic frontiers (**Table 2**).

**Table 2:** Comparative analysis of therapeutic strategies: clinical status, risks, and benefits.

Category	Intervention	Clinical status	Primary benefits	Risks and limitations
Conventional	Behavioral (CBIT/ERP)	First-line treatment	High safety profile with no medical side effects; efficacy comparable to medication in some trials	Highly dependent on trained therapists; limited accessibility; less effective for those with severe anxiety
	Pharmacological (Aripiprazole)	Standard clinical practice for moderate to severe cases	Rapid and reliable symptom control for motor and vocal tics.	Common side effects include weight gain, metabolic shifts, and extrapyramidal symptoms.
Immune-Targeted	Antibiotics (Penicillin/Azithromycin)	Clinical practice for identified PANDAS subgroup	Eradicates acute GABHS infection, addressing the potential trigger	Potential for antibiotic resistance; specific cardiac risks associated with Azithromycin.
	IVIG/Plasmapheresis (PEX)	Experimental/Reserved for refractory/severe cases	Modulates complex immune responses; rapid reversal of symptoms in acute-phase PANS/PANDAS	Invasive; risk of thrombosis, aseptic meningitis, and central venous catheter complications.
	Corticosteroids (Prednisone)	Not mainstream; short-term rescue therapy	Temporary suppression of acute inflammatory flares	Significant risks including growth suppression, bone density loss, and potential to worsen tics
Emerging/Future	FMT/Probiotics	Theoretical/Exploratory research stage.	Targets the gut-microbiome-brain axis to restore long-term homeostasis	Lack of standardized protocols; long-term efficacy and safety profiles remain unverified
	Targeted B-cell Therapy (Rituximab)	Experimental; specific autoimmune subtypes only	Precisely eliminates B-cells responsible for pathogenic autoantibodies	Severe immunosuppression; increased susceptibility to life-threatening infections

Behavioral therapy is currently the internationally recognized first-line non-pharmacological treatment. According to guidelines from the American Academy of Neurology (AAN) and the European Society for the Study of Tourette Syndrome (ESSTS), Comprehensive Behavioral Intervention for Tics (CBIT) has been proven effective for tic symptoms in both children and adults (69, 70). Notably, CBIT is the only therapy to receive a “high confidence” efficacy rating in the AAN guidelines and is typically recommended before trying medication or other interventions. Multiple studies show that CBIT can reduce scores on the Yale Global Tic Severity Scale (YGTSS) by 26-31%, and one randomized controlled trial (RCT) indicated its efficacy is comparable to medication (71, 72). The core techniques of CBIT include Habit Reversal Training (HRT) to increase awareness of premonitory urges and Competitive Response Training to suppress tics (42). Additionally, Exposure and Response Prevention (ERP) is an effective strategy aimed at increasing tolerance to premonitory urges to reduce tic occurrence. To improve accessibility, telemedicine and video conferencing have been shown to be as effective as face-to-face therapy, while group therapy offers a more cost-effective option (73).

Despite the significant effectiveness of behavioral therapy, its application still faces challenges. First, its efficacy depends on well-trained therapists, and uneven resource distribution limits its accessibility. Second, some patients may benefit less due to comorbidities (such as severe anxiety) or strong premonitory urges. A data analysis pointed out that while ADHD or OCD itself does not affect CBIT’s efficacy, co-existing anxiety disorders and severe premonitory urges are associated with a poorer treatment response. This analysis also suggested that patients currently using tic-suppressing medication might have a less effective response to CBIT than those not on medication, but further research is needed on the optimal intervention age and the interactive effects of different drugs.

Pharmacological therapy is an important option for patients with moderate to severe symptoms. However, the AAN guidelines note that due to a lack of sufficient large-scale, high-evidence-level clinical trials, no single medication has received a “high confidence” rating for its efficacy in reducing tics. The ESSTS guidelines, based on existing evidence and expert consensus, propose a hierarchical approach to drug selection, with aripiprazole being the most commonly used option across all age groups. Although drugs can be very successful in controlling symptoms, insufficient efficacy or intolerable adverse effects (such as weight gain, metabolic disorders, extrapyramidal symptoms) are common clinical dilemmas (74).

These limitations have driven the exploration of novel pharmacological compounds, but with mixed results. The dopamine D1 receptor antagonist ecopipam showed good efficacy and safety in a small, double-blind RCT but has not yet been included in mainstream guidelines (75). Vesicular monoamine transporter 2 (VMAT2) inhibitors, such as valbenazine and deutetrabenazine, showed potential in early open-label trials but failed to demonstrate significant efficacy over placebo in subsequent placebo-controlled Randomized Controlled Trials (RCTs) (76). The use of cannabinoids is still in the experimental stage. While some retrospective data and open-label studies have reported symptom improvement, multiple systematic reviews and meta-analyses have failed to confirm a reliable tic-suppressing effect (77). Preliminary results from a double-blind RCT on nabiximols also did not show superiority. Therefore, both AAN and ESSTS guidelines classify them as experimental treatments for refractory patients, and their clinical use requires extreme caution (78).

In summary, while conventional treatments have their value, their limitations (including the accessibility and influencing factors of behavioral therapy, and the efficacy-safety balance of pharmacological treatment) clearly point to the urgent need for developing more precise and safer innovative therapies, especially those targeting the underlying immunopathological mechanisms (76).

### 4.2 Treatment targeting the infectious source

In the PANDAS subgroup, where tic disorders are associated with GAS infections, treating the infectious source is a core strategy. Antibiotic therapy is primarily used to eradicate an acute GAS infection. In clinical practice, a course of antibiotics, such as penicillin or azithromycin, is recommended for PANDAS children with confirmed GAS pharyngitis to eliminate the pathogen (79, 80). Some studies have shown that azithromycin can also help alleviate PANDAS symptoms; however, due to its potential side effects, mainly cardiac risks, its use should be strictly controlled. The practice of using prophylactic antibiotics to prevent GAS recurrence and thus prevent tic exacerbations remains highly controversial. While some open-label studies and case reports have shown positive effects, there is a lack of high-quality RCTs to confirm their long-term efficacy and safety. A systematic review pointed out that the quality of evidence supporting the routine use of antibiotics in the absence of an active infection is low, and more research is needed to clarify the benefit-risk ratio. Therefore, the prophylactic use of antibiotics should be an individualized decision, with full consideration of the potential risk of antibiotic resistance (42).

### 4.3 Core immunomodulatory therapies

A growing body of evidence suggests that immune dysregulation plays a key role in the pathogenesis of some pediatric tic disorders, particularly in infection-associated subtypes like PANS/PANDAS (81). Therefore, therapies targeting the immune system, known as immunomodulatory therapies, have become an important strategy for treating severe or refractory cases (82). These therapies aim to suppress or modulate abnormal immune responses to alleviate neuropsychiatric symptoms (83). This section will focus on three core immunomodulatory therapies: IVIG plasma exchange (PEX), and corticosteroids.

#### 4.3.1 Intravenous immunoglobulin

Intravenous immunoglobulin is an antibody preparation pooled, purified, and concentrated from the plasma of thousands of healthy donors. Its mechanism of action in autoimmune neurological diseases is multidimensional and complex (84). First, IVIG provides a broad spectrum of neutralizing antibodies that can recognize and eliminate potential infectious pathogens and neutralize circulating pathogenic autoantibodies, including those that may target basal ganglia neurons (85). Second, IVIG exerts powerful immunomodulatory effects by interacting with Fcγ receptors (FcγR). This interaction triggers several mechanisms: it inhibits complement activation, downregulates pro-inflammatory cytokines (IL-1, TNF-α), and promotes anti-inflammatory cytokines like IL-10. Additionally, it suppresses the activation of antigen-presenting cells (86). Furthermore, studies have shown that IVIG can regulate the functional balance of T cells and B cells. For example, it can induce the expansion of regulatory T cells (Tregs) and inhibit the proliferation and antibody production of autoreactive B cells, thereby re-establishing immune homeostasis (87).

IVIG is considered a first or second-line immunomodulatory therapy for PANS/PANDAS and other potentially immune-mediated tic disorders, especially for moderate to severe patients who respond poorly to standard treatments (like antibiotics and cognitive-behavioral therapy). Multiple open label studies and retrospective analyses have shown that children treated with high-dose IVIG (typically 1-2 g/kg infused over 1-2 days) achieve significant improvements in tics, obsessive-compulsive symptoms, and other neuropsychiatric symptoms (81–88). A study of 65 PANDAS children found that after a single IVIG infusion, 20% had complete symptom remission, and 65.5% achieved long-term symptom improvement after needing a second infusion, with the effect lasting at least one year (23). Although large-scale RCTs are lacking, existing evidence supports the effectiveness of IVIG in specific patient populations, especially when applied early in the disease course.

IVIG therapy is generally well-tolerated, but adverse reactions can occur. Common side effects are usually related to the infusion rate, are mild, and self-limiting, including headache, fever, chills, nausea, and fatigue (89). These reactions can often be prevented or managed by slowing the infusion rate or using antipyretics and antihistamines. Rarer but more serious side effects include aseptic meningitis, renal impairment, hypertension, and thromboembolic events. Therefore, it is crucial to ensure patients are well-hydrated during IVIG treatment and to closely monitor patients with high-risk factors (such as a hypercoagulable state or a history of cardiovascular disease).

#### 4.3.2 Plasma exchange

Plasma exchange (or plasmapheresis) is an extracorporeal procedure that rapidly removes large molecules from the plasma by separating the patient’s plasma from their blood cells, discarding it, and replacing it with a substitute fluid (such as albumin or fresh frozen plasma) (90). Its core therapeutic mechanism is the efficient removal of circulating pathogenic autoantibodies, immune complexes, complement components, and various inflammatory factors, which are considered the direct mediators of autoimmune damage to the nervous system.

In the field of tic disorders, PEX is mainly used for critically ill patients, those with explosive symptom exacerbations, or refractory cases that do not respond to other immunotherapies like IVIG, especially during the acute phase of PANS/PANDAS (91). An early randomized controlled trial showed that PEX was superior to IVIG and placebo in improving tic and obsessive-compulsive symptoms, with a particularly rapid onset of action in the short term (92). Although the procedure is relatively complex, requires specialized medical centers, and carries risks associated with central venous catheterization (such as infection and bleeding), PEX remains an effective treatment option that can rapidly reverse the condition for patients facing severe functional impairment.

#### 4.3.3 Corticosteroids

Corticosteroids are potent, non-specific anti-inflammatory and immunosuppressive agents. Their mechanism of action is broad; by binding to glucocorticoid receptors, they regulate the transcription of numerous genes, thereby inhibiting the synthesis of multiple pro-inflammatory cytokines and chemokines, reducing the activation, proliferation, and migration of immune cells (such as lymphocytes and macrophages), and inducing lymphocyte apoptosis, thus comprehensively suppressing immune system function (93). However, the use of corticosteroids in pediatric tic disorders remains controversial, and the evidence is limited. Some small-scale studies and case reports suggest that short-term oral or pulse doses of corticosteroids (such as prednisone) may lead to a temporary improvement in tic symptoms in some children (88). However, some research indicates that while steroids may improve obsessive-compulsive symptoms, they can conversely worsen tics, and symptoms are highly likely to relapse after discontinuation. More importantly, long-term or high-dose use of corticosteroids carries significant risks and side effects, especially in children and adolescents. These risks include growth suppression, decreased bone density, Cushing’s syndrome, hyperglycemia, hypertension, mood and behavioral changes (such as irritability and depression), and increased susceptibility to infections (93, 94). Given these potential serious adverse effects and the uncertainty of their efficacy, corticosteroids are not currently a mainstream treatment for pediatric tic disorders. They are usually only considered as a short-term “bridging” or rescue therapy in very specific circumstances after a careful risk-benefit assessment.

### 4.4 Emerging and future therapies

Emerging immunomodulatory therapies offer new directions for the treatment of pediatric tic disorders, with a core focus on intervening in the immune dysregulation and neuroinflammatory processes associated with pathogenesis. Among these, regulating the gut-brain axis function shows particularly broad prospects. Studies indicate that specific strains of probiotics may exert their effects by modulating the immune system, reducing intestinal permeability, and influencing neurotransmitter metabolism. A preliminary clinical trial showed that a specific probiotic intervention helped improve tic symptoms. A preliminary clinical study confirmed that FMT, by remodeling the gut microbiota composition, can significantly reduce tic symptoms in children (95, 96). This mechanism has also been validated in animal models, showing that FMT can modulate gut microbiota to influence neurotransmitter secretion, thereby improving tic-like behaviors (95, 97).

Another more precise strategy is targeted B-cell therapy, which aims to eliminate B lymphocytes that may produce pathogenic autoantibodies. Although direct evidence is lacking in typical tic disorders, in severe cases of the related pediatric autoimmune neuropsychiatric disorders (PANDAS), a combination therapy including rituximab has been successfully reported, suggesting its potential value in specific autoimmune-driven subtypes (98, 99). Furthermore, the application of small-molecule immunomodulators offers new ideas. For example, a case report showed that the selective COX-2 inhibitor celecoxib could continuously improve a patient’s tics and related behavioral problems, providing direct evidence for the involvement of inflammatory pathways in the pathophysiology of tic disorders (100).

In summary, evidence from gut microbiota modulation, B-cell targeted therapy, and small-molecule anti-inflammatory drugs collectively highlights the feasibility and potential of targeting the immune system for the treatment of tic disorders. However, larger-scale randomized controlled trials are urgently needed to further validate their clinical efficacy and safety. Such treatments should be strictly confined to professional medical centers with scientific research qualifications and must undergo rigorous ethical review.

## 5 Discussion

This review has systematically delineated an increasingly pivotal dimension in the pathophysiology of pediatric Tic Disorders (TDs): immune dysregulation. While the traditional dopamine-centric theory provides a foundational framework for understanding the dysfunction of the cortico-striato-thalamo-cortical (CSTC) circuitry, it falls short of explaining the complex clinical heterogeneity observed in the full spectrum of TDs. Specifically, the classical model cannot fully account for the waxing and waning nature of symptoms, the phenomenon of spontaneous remission in adolescence, and, most importantly, the subset of patients who present with acute, explosive symptom onset triggered by environmental factors such as infections. The evidence synthesized in this review suggests that an immune-mediated model should not be viewed as a universal explanation for all tic disorders, but rather as a core pathogenic mechanism for a distinct clinical subgroup particularly those fitting the PANDAS/PANS profile where immune dysfunction acts as a critical driver of neurochemical imbalance.

Moving beyond isolated findings, we propose a hypothetical integrative framework where autoimmunity, neuroinflammation, and gut-brain axis dysfunction act as synergistic, interconnected pathways. In this theoretical model, we hypothesize a “multi-hit” process: First, upstream gut dysbiosis may act as a persistent source of systemic low-grade inflammation and altered neurotransmitter precursors (such as GABA), priming the developing immune system and rendering the CNS more vulnerable (101, 102). Second, upon exposure to specific pathogens (GABHS), molecular mimicry may trigger the production of autoantibodies that cross-react with neuronal targets in the basal ganglia. Crucially, for these peripheral antibodies to exert pathogenic effects on the CNS, a “gateway” must be opened; this is likely facilitated by neuroinflammation specifically, Th17 cell-mediated disruption of the blood-brain barrier (BBB). This breach allows autoantibodies and inflammatory cytokines to penetrate the CNS, directly altering synaptic transmission and dopamine receptor sensitivity within the CSTC circuit, ultimately manifesting as motor and vocal tics. While individual components of this cascade such as microglial activation observed in PET studies or specific gut microbiota alterations have experimental support, it is important to emphasize that the complete, sequential chain of events from gut dysbiosis to antibody penetration and tic generation remains a theoretical construct that requires further validation in human longitudinal studies.

However, translating this immunopathological framework into clinical practice is currently hindered by several critical limitations and inconsistencies in the available literature. First, the reproducibility of immunological biomarkers is variable. Findings regarding serum cytokine profiles and anti-neuronal antibody titers (including Anti-D1R/D2R and CaMKII activation) have been inconsistent across different cohorts. This heterogeneity likely stems from methodological differences (ELISA vs. cell-based assays), small sample sizes, and the fluctuating nature of the disease itself, where immune markers may normalize during remission periods. Consequently, no single immune biomarker has yet been validated for routine clinical diagnosis. Second, regarding the microbiome, the direction of causality remains a significant point of debate. While gut dysbiosis is frequently observed in patients with TDs, it is currently unclear whether these alterations are a primary driver of pathogenesis or secondary consequences of the disorder. Confounding factors such as selective dietary habits associated with sensory sensitivities, psychological stress, and, most notably, the use of pharmacological treatments (antipsychotics like aripiprazole) can profoundly alter the gut microbiota. Most existing studies are cross-sectional and fail to control for these variables, making it difficult to distinguish cause from effect. Third, a substantial portion of the mechanistic evidence, such as the passive transfer of antibodies inducing stereotypes, relies on murine models. However, significant differences exist between rodents and humans regarding immune system development, microbiome composition, and blood-brain barrier maturation. These biological discrepancies limit the direct translation of findings from animal models to pediatric patients and may explain why some treatments effective in mice fail in human clinical trials. Finally, the clinical entities of PANDAS and PANS remain subjects of academic debate. The current diagnostic criteria rely heavily on clinical history and the temporal association with infections. This approach is inherently subjective and prone to recall bias, leading to potential overdiagnosis or misdiagnosis. The lack of a universal consensus on the precise diagnostic boundaries between “classic” Tourette Syndrome and these immune-mediated subtypes further complicates patient stratification for research.

Despite these uncertainties, the recognition of immune involvement has catalyzed a paradigm shift towards novel therapeutic strategies that move beyond symptomatic neurotransmitter modulation. Emerging interventions targeting the root causes of immune dysregulation are showing promise. FMT aims to restore gut homeostasis, potentially dampening systemic inflammation from the source. Targeted B-cell therapies (rituximab) seek to eliminate the production of pathogenic autoantibodies, while immunomodulatory agents like IVIG attempt to neutralize circulating antibodies and regulate immune responses. Preliminary reports and case studies suggest that these interventions may offer profound benefits for patients with refractory, severe, or clearly infection-associated symptoms who do not respond to conventional therapies. However, a note of extreme caution is warranted. These immunomodulatory therapies are currently experimental and are not recommended as standard clinical options. Unlike behavioral therapy or standard tic-suppressing medications, these interventions carry profound risks, including severe immunosuppression, susceptibility to serious infections, and potential metabolic disturbances. Furthermore, the current evidence base is largely derived from open-label studies or small case series, which are susceptible to the placebo effect and publication bias. In conclusion, while the immune hypothesis offers a compelling explanation for a subset of Tic Disorders, the field must now transition from exploratory observation to rigorous validation. Future research must prioritize large-scale, double-blind RCTs to definitively establish the safety and efficacy of emerging immunotherapies. Simultaneously, prospective longitudinal cohort studies utilizing multi-omics approaches are urgently needed to clarify the causal relationships within the neuroimmune network and to identify stable biomarkers that can reliably stratify patients for precision medicine.

## 6 Conclusion

In conclusion, the pathophysiological understanding of pediatric Tic Disorders is evolving from a strictly dopaminergic model to a more comprehensive framework that incorporates neuroimmunological dimensions. Current evidence suggests that immune dysregulation spanning autoimmunity, neuroinflammation, and gut-brain axis imbalances is a key pathogenic driver. However, this likely applies to a specific subset of patients, such as those with PANS/PANDAS, rather than the general TD population.

While the integration of multi-dimensional data from serology, cerebrospinal fluid, and neuroimaging holds promise for identifying immune-related biomarkers and achieving patient stratification, these tools require further validation to ensure their reproducibility and clinical utility. Furthermore, innovative immunomodulatory interventions, including FMT and targeted B-cell therapies, represent potential frontiers for children who respond poorly to conventional treatments. However, it is imperative to recognize that these strategies are currently experimental. Large-scale, high-quality RCTs are essential to definitively establish their long-term safety and efficacy before they can be integrated into routine clinical practice. Ultimately, continued exploration of these immune mechanisms, balanced with rigorous methodological validation, will be key to advancing personalized diagnosis and treatment for affected children.
